# Relative aerobic load of daily activities in individuals with lower limb amputation: A pilot study

**DOI:** 10.33137/cpoj.v8i2.46249

**Published:** 2025-12-31

**Authors:** L. van Schaik, K. van Kammen, S.W.M. Huiberts, J.H.B. Geertzen, H. Houdijk, R. Dekker

**Affiliations:** 1 University of Groningen, University Medical Center Groningen, Department of Rehabilitation Medicine, Groningen, The Netherlands.; 2 University of Groningen, University Medical Center Groningen, Department of Human Movement Sciences, Groningen, The Netherlands.

**Keywords:** Relative Aerobic Load, Lower Limb Amputation, Activities of Daily Living, First Ventilatory Threshold, Amputation

## Abstract

**BACKGROUND:**

Individuals with lower limb amputation (LLA) often experience reduced physical capacity and increased aerobic demands during activities of daily living (ADL), which may impact participation and quality of life. Measuring the relative aerobic load of ADL is essential to set realistic rehabilitation goals, however data on short and non-steady-state ADL tasks in this population are limited.

**OBJECTIVES:**

To evaluate the feasibility of a newly developed protocol for assessing the relative aerobic load of five selected ADL tasks in individuals with LLA, and to determine the applicability of the excess post-exercise oxygen consumption (EPOC) method for these measurements.

**METHODOLOGY:**

Six individuals with unilateral LLA (mean age 59 ± 9 years; five males), including three with transtibial, one with knee disarticulation, and two with transfemoral amputation, all classified as K2–K3 ambulators, were recruited for this pilot study and underwent a cardiopulmonary exercise test (CPET) during the first assessment session. During the second assessment session, within a two-week interval, participants were asked to perform five standardized tests reflecting ADL tasks: the 6-Minute Walk Test (6MWT), Timed Up and Go Test (TUGT), Glittre ADL test, stair ascent, and stair descent. Oxygen consumption was measured using the COSMED K5 system. For each task, average oxygen consumption was computed as the total oxygen uptake during activity and recovery (EPOC) divided by total time, and relative aerobic load was defined as percentage of first ventilatory threshold (%${\dot{V}O}_{2}$-VT_1_). Data analysis involved descriptive feasibility assessment and intraclass correlation to examine consistency of relative aerobic load across tasks.

**FINDINGS:**

The protocol showed to be feasible and no (serious) adverse events occurred during the study and 5 out of 6 participants were able to complete the protocol, demonstrating its practicality. The EPOC method was successfully applied to both short and longer-duration ADL tasks. The results showed relative aerobic loads exceeding 100% ${\dot{V}O}_{2}$-VT_1_ for most ADL tasks, indicating substantial physical effort. Agreement of the rankings of the different tasks between participants was observed (intraclass correlation coefficient (ICC) = 0.73, 95% confidence interval (CI) = 0.01–0.97), although the wide confidence interval indicates considerable uncertainty.

**CONCLUSION:**

This protocol enables the assessment of relative aerobic load in a heterogeneous group of individuals with LLA and highlights the importance of individualized assessment. Given the large variability in ${\dot{V}O}_{2}$peak and ${\dot{V}O}_{2}$-VT_1_ between participants, a generic level of physiological burden for each task cannot be given, instead individualized assessment is crucial to accurately determine physiological burden and avoid under- or overestimation of exercise intensity. The findings support the development of individualized rehabilitation programs tailored to individual capabilities and limitations, potentially improving participation and quality of life. Additionally, future studies with larger cohorts are needed to provide more robust reliability estimates and strengthen the generalizability of these findings.

## INTRODUCTION

Participation in societal activities requires a certain level of physical capacity to perform activities of daily living (ADL). Individuals with lower limb amputation (LLA) typically exhibit reduced physical capacity compared to able-bodied individuals,^1,2^ and walking with a prosthesis requires more energy, resulting in a higher relative aerobic load.^3–5^ A higher relative aerobic load indicates that an activity demands a greater proportion of an individual's aerobic capacity, which may lead to increased fatigue and reduced tolerance for sustained activity. This elevated aerobic load is likely not limited to walking alone, but may also apply to other ADL. Consequently, individuals with LLA may experience increased fatigue, perform at slower walking pace,^6–11^ or avoid certain activities altogether, which can negatively impact their ability to perform ADL, their quality of life^12,13^ and their societal participation.^14,15^ To set realistic rehabilitation goals for individuals with LLA, it is essential to understand the relative aerobic load for various ADL tasks in relation to the individual physical capacity. During a cardiopulmonary exercise test (CPET), peak oxygen uptake (${\dot{V}O}_{2}$peak) can be assessed as an indicator of maximum aerobic capacity.^16^ Additionally, oxygen uptake at the first ventilatory threshold (${\dot{V}O}_{2}$-VT_1_) can be determined as an indication of anaerobic threshold.^16^ Previous studies in other populations suggest that ADL with oxygen consumption level below the ${\dot{V}O}_{2}$-VT_1_ can be sustained for longer durations. The relative aerobic load (i.e. the aerobic load as percentage of ${\dot{V}O}_{2}$-VT_1_) can be used to express the degree of effort required per activity.^17,18^

While ADL tasks such as stair climbing and meal preparation are important for individuals with LLA,^19^ the relative aerobic load associated with these activities is largely unexplored, leaving a significant gap in current knowledge. A recent study found that individuals with LLA (14 transtibial and 7 transfemoral) exhibited a higher relative peak oxygen uptake (%${\dot{V}O}_{2}$peak) during in-house walking compared to able-bodied controls (n = 12).^20^ However, the study primarily included high-functioning participants, limiting generalizability. Moreover, data on short-duration ADL beyond walking are lacking, possibly due to methodological challenges in assessing non-steady-state activities. These include ADL tasks such as stair climbing, meal preparation, as well as brief movements like standing up to retrieve an item, carrying something away, or opening a door.

In an effort to address the methodological challenges, the excess post-exercise oxygen consumption (EPOC) method was recently developed and applied in individuals post-stroke,^18^ offering a way to measure the relative aerobic load of short-duration ADL. This method is capable of measuring the relative aerobic load of short ADL in individuals post-stroke. However, there is currently no standardized protocol to investigate the relative aerobic load in individuals with LLA. The LLA population is highly diverse in terms of general fitness and comorbidity, with comorbid conditions themselves further influencing physical capacity. Given the heterogeneity of the LLA population in the Netherlands, where most people are older adults with multiple comorbidities,^21^ it is crucial to select ADL tasks that are representative of this group's functional capabilities. However, standardized protocols to evaluate the relative aerobic load of these tasks in this population are lacking.

The aim of this pilot study was to assess the developed protocol based on various criteria related to operability and logistics, as well as its suitability in terms of intensity, ensuring that individuals with LLA can effectively perform and complete the selected ADL. Additionally, the study assessed whether the EPOC method can be effectively applied to determine the relative aerobic load of five different ADL tasks, both short and longer in duration.

## METHODOLOGY

This pilot study had an observational design. Informed consent was obtained from each participant prior to testing. The Medical Ethical Committee of the University Medical Centre Groningen (UMCG) has approved this study (METc nr: NL2018000572). This study was performed in accordance with the Declaration of Helsinki.^22^

### Participants

Six individuals with LLA were recruited through flyers distributed in the waiting rooms of the orthopedic center in Haren, the Roessingh Orthopedic Center (Enschede), and the amputation rehabilitation unit at the UMCG Centre for Rehabilitation, Beatrixoord. Interested people contacted the researchers (LvS/SH) directly. Recruitment took place from late October 2023 until early February 2024. It is unknown how many people took a flyer, but all individuals who contacted the researchers met the inclusion criteria. The study protocol was designed for six participants; however, one participant withdrew from the study due to illness, and a seventh participant was recruited as a replacement. After six participants were successfully included and completed the study, recruitment was closed. Inclusion criteria were: (**1**) Participants had undergone unilateral LLA (transtibial, knee disarticulation or transfemoral) over one year prior to participation, (**2**) the causes of LLA to be either vascular, trauma, infection or cancer, (**3**) participants using their own prosthesis for daily functioning and (**4**) had an activity level of K2 (ability to traverse low-level environmental barriers) or K3 (ability for ambulation with variable cadence). Participants were excluded if: (**1**) were not eligible to perform CPET according to the American College of Sports Medicine guidelines,^23^ (**2**) suffered from severe psychiatric illness, or (**3**) had residual limb problems that limited prosthesis use at the time of inclusion. The researcher (LvS) discussed contraindications for CPET (e.g., recent cardiovascular event, acute pulmonary embolism, systolic blood pressure >200 mmHg or diastolic >120 mmHg) with participants when they expressed interest in joining the study. This was conducted in accordance with the existing protocol implemented as standard care for screening individuals prior to CPET at the UMCG Centre for Rehabilitation, Beatrixoord.

### Five Selected ADL Tasks

The ADL tasks in this study were chosen based on two criteria: **(1)** standardized tests commonly used in LLA rehabilitation programs and **(2)** priorities identified by individuals with LLA in a previous questionnaire study.^19^

The selected tests represent key domains of daily functioning, including standing up and walking short distances, walking longer distances, ascending and descending stairs, and performing complex functional activities that simulate household tasks.

The Timed Up and Go Test (TUGT) was used to assess short-distance walking,^24^ while the 6-Minute Walk Test (6MWT) evaluated longer-distance walking.^25^ Stair ascent and descent were performed by walking through a hallway and then ascending and descending a staircase with 21 steps, ensuring the activity lasted more than three minutes to simulate a realistic ADL scenario. The Glittre ADL test is a widely used timed performance test that evaluates functional capacity through five laps of a 10-meter circuit (total 50 meters). The circuit incorporates multiple functional movements such as sitting and standing from a chair, walking, going up and down a few steps, reaching, squatting, turning, and moving object. This test reflects complex daily activities like tidying up the house or putting away groceries and has been validated in populations with chronic obstructive pulmonary disease and heart failure.^26–29^

### Cardiopulmonary Exercise Test

The one-leg ergometer (Ergoselect 200-Ergoline) was used for the CPET, which has previously been validated for individuals with LLA^30–32^ for determining rest ${\dot{V}O}_{2}$, ${\dot{V}O}_{2}$-VT_1_ and ${\dot{V}O}_{2}$peak.

### Procedures

The participants were tested at the UMCG Centre for Rehabilitation, Beatrixoord. The sessions were divided over two days within a two-week interval. The first assessment session started with a baseline assessment containing: Medical intake, short physical examination, measuring rest metabolism and performing a CPET#. During the CPET a certified clinical exercise physiologist and/or physician was present.

The second assessment session took place within 14 days after first assessment session and began with a short review to check for any physical discomfort or issues following the CPET. Each participant then performed the five ADL tasks in the same order: 6-Minute Walk Test (6MWT), Timed Up and Go Test (TUGT), Glittre ADL test, stair descent, and stair ascent. Participants P01, P03, and P05 completed these tasks in the morning (09–12), while P02, P04, and P06 performed them in the afternoon (13–17). Before the first task (6MWT), a 10-minute rest measurement was taken to establish baseline values. After each task, measurements continued for 10 minutes to return to baseline, followed by a 30-minute break. Each subsequent task began with a 2-minute rest measurement. After completing all tasks, participants completed a questionnaire evaluating feasibility and their experience.

During all ADL tasks, breath-by-breath gas exchange was measured using the COSMED K5b system (CE MED-9811). The TUGT was repeated three times to provide a more reliable estimate of the energy requirement for this relatively short test. The duration of the ADL tasks ranged between 3 and 6 minutes, except for the repeated TUGT, which lasted approximately 7–8 minutes due to the additional rest periods.

### Study Outcomes

Data to determine feasibility of the protocol was collected based on pre-set feasibility criteria as described in **Table 1**. During the CPET the following data was collected; ${\dot{V}O}_{2}$ rest (ml/kg/min), ${\dot{V}O}_{2}$-VT_1_, ${\dot{V}O}_{2}$peak (ml/kg/min), maximum heartrate (beats/min), respiratory exchange ratio (RER), maximum power (W) and % predicted heartrate.

**Table 1:** Feasibility criteria of the protocol.

Feasibility Criteria	Outcome (yes/no)
**1.** For 5 out of 6 of the participants the breaks are adequate in terms of length and frequency (to return to rest metabolism)	Yes^*^ (6/6)
**2.** 5 out of 6 the participants can complete the entire protocol	Yes^**^ (5/6)
**3.** The logistics are adequate: 6/6 participants will receive the time schedule at least two weeks in advance It is clear for 6/6 participants who he/she can call for questions (e.g. about the schedule) When arriving at the UMCG Centre for Rehabilitation, Beatrixoord, it is clear for 6/6 participants how and where he/she can register It is clear for 6/6 participants where they have to wait before the tests No issues with planning to arrange all test moments For 6/6 participants there is a clear end of the testing day with a short evaluation moment with the participant and researcher The schedule of the test day does not exceed more than 30 minutes due to logistical causes (not based on participant related causes)	*Yes (6/6)* Yes^*^ (6/6) Yes^*^ (6/6) Yes^*^ (6/6) *Yes (6/6)* *Yes (6/6)* *Yes (6/6)*
**4.** No essential data are missing for the analysis, conform previous described study parameters	*Yes (6/6)*

* Based on responses of participants asked after the second testing day.

** Adjusted version of the Glittre ADL was needed for three participants to ensure everybody could perform the test; participants walked the same distance but on a level surface instead of steps.

### Data Analysis and Statistics

Feasibility was assessed using a pre-defined evaluation form based on the criteria shown in **Table 1**. Peak aerobic capacity (expressed as ${\dot{V}O}_{2}$peak) was determined from the CPET data. The ${\dot{V}O}_{2}$peak was considered maximal when the participant reached an RER > 1.1 and/or predictive heart rate > 85%. The timepoint of ${\dot{V}O}_{2}$-VT_1_ was determined with the V-slope method,^33^ by two certified clinical exercise physiologists (ML/MH), working independently and not involved in other aspects of the study conform the method described in a previous study.^18^ When the difference of the ${\dot{V}O}_{2}$-VT_1_ between assessors was more than 100 ml.O_2_/min, they conferred for consensus. The average rate of oxygen consumption of each ADL task was calculated as the sum of oxygen consumption (O_2_ml/kg) during the ADL tasks and excess post exercise consumption phase, divided by the time to complete the activity as previously outlined in detail by Blokland et al.^18^ The relative aerobic load was defined as the average oxygen consumption divided by ${\dot{V}O}_{2}$-VT_1_, expressed as % ${\dot{V}O}_{2}$-VT_1_

The intraclass correlation (ICC, two-way mixed design with type consistency) was calculated to assess the correlation of relative aerobic load (% ${\dot{V}O}_{2}$-VT_1_) on different ADL tasks between participants,^34^ to assess the consistency in ranking of the participants in this heterogeneous group between ADL tasks. Analyses were performed using IBM SPSS Statistics, version 28.

## RESULTS

Six participants were included in the study (**Table 2**). In total, seven people contacted the researchers and were screened; all met the inclusion criteria, but one withdrew due to illness, resulting in the inclusion of 6 participants. This number was sufficient, as only six participants were required for the study. Mean age was 59 ± 9 years, five males, one female. Three participants with transtibial amputations (TTA), one with knee disarticulation and two participants with transfemoral amputations (TFA) were included. Four participants with LLA due to vascular reasons, the other two due to infections of total knee prosthesis. Within this group, a range of comorbidities was present.

**Table 2:** Participant characteristics.

Participant #	01	02	03	04	05	06
**Amputation Level**	TTA	TTA	TFA	TTA	KD	TFA
**Age (y)**	61.3	45.6	57.7	53.5	71.8	64.7
**Sex (m/f)**	M	M	M	M	M	F
**Years Since Amputation**	1.3	5.9	3.2	1.1	12	2.7
**Walking Aids, Besides Prosthesis**	No	Yes, walker and AFO	No	No	Yes, two crutches	Yes, walker and AFO
**Type of Prosthesis**	Passive vacuum with sleeve; Össur liner; Ottobock Taleo foot	Shuttle lock (pin system); Össur Synergy liner; Össur Pro-flex XC foot	Passive vacuum with anatomically shaped socket; Seal-In liner; C-Leg (microprocessor knee); Össur Flex-Foot	Passive vacuum system with sleeve; Össur Dermo liner; Ottobock Taleo foot	Femoral condyle fit socket with a polyform insert; Össur Total Knee 1900; Ottobock Trias foot	IRC socket with passive vacuum system; movable Seal-In ring (Össur liner); Össur Total Knee 2100; Össur Vari-flex foot
**Height (cm)**	182	165	185	185	172	173
Weight (kg)^*^	85	79	91	94	88	122
**K-Level**	K3	K2	K3	K3	K2	K2
**Cause of Amputation**	Vascular	Vascular	Infection	Vascular	Vascular	Infection
**Comorbidities**	CVD	CVD, stroke	CVD, sarcoidosis	CVD, DM2	CVD, COPD, DM2, HTN	Asthma, HNP L4-L5 with drop foot
**Use Beta Blockers**	No	No	No	Yes	Yes	No
**Smoke**	No	No	No	Yes	Yes	No
**Berg Balance Scale**	56	48	53	56	48	44

TTA= Transtibial Amputation; KD = Knee Disarticulation; TFA = Transfemoral Amputation; AFO = Ankle Foot Orthosis; CVD = Cardio Vascular Disease; DM2 = Diabetes Mellitus Type 2; COPD = Chronic Obstructive Pulmonary Disease; HTN = Hypertension; HNP = Herniated Nucleus Pulposus.

* Weight is without prosthesis.

### Feasibility

All feasibility criteria of the protocol were met. Of the six participants, five successfully completed all tasks. Participant 06 did not complete all tasks.

Modifications to the Glittre ADL test were required for three participants (P03, P05, and P06) due to the use of walking aids and the absence of support bars (i.e., railings or other stable structures for hand support) to enhance stability. These adaptations were necessary because the steps could not be safely combined with the participants' own walking aids, and no railings or walls were available for additional support. To ensure safety and create a testing environment that felt secure for participants, while simulating a realistic daily situation using their own walking aid, the protocol was adjusted. In the modified version, participants walked the same distance on a level surface instead of steps. One participant (P06) was unable to complete the stair tasks due to fear of the unfamiliar staircase and therefore did not complete the full protocol. All participants reported that the breaks were sufficient and that the logistics were well organized, with clear instructions and communication. All necessary data for analysis were successfully collected.

### Relative Aerobic Load for ADL Tasks

The CPET data are presented in **Table 3**. One participant stopped the CPET within warming up, because this participant couldn't cycle well with one leg due to coordination problems, therefore this participant was excluded from further analysis. Of the remaining five participants, only one achieved a maximum CPET based on RER and/or predicted heart rate. The mean ${\dot{V}O}_{2}$ at rest was 4.1 ± 0.5 ml/kg/min, mean ${\dot{V}O}_{2}$-VT_1_ was 10.0 ± 2.4 ml/kg/min. Stop reasons were because of fatigue in the leg and/or shortness of breath.

**Table 3:** Cardiopulmonary exercise test (CPET) data for each participant.

Participant	01	02	03	04	05	06
**${\dot{V}O}_{2}$ in Rest (ml/kg/min)**	4.7	4.7	3.5	3.5	4.1	N/A
**${\dot{V}O}_{2}$ at VT_1_ (ml/kg/min)**	7.9	10.0	13.3	7.6	11.3	N/A
**${\dot{V}O}_{2}$peak During Test (ml/kg/min)**	16.6^*^	11.5	17.0	8.5	12.1	N/A
**RER**	1.27	0.94	1.01	0.99	0.95	N/A
**Watt Peak (W/kg)**	1.1	0.4	1.0	0.3	0.6	N/A
**Heart Rate peak**	148	122	98	95	92	N/A
**% Predicted HR**	93	70	60	57	62	N/A

* Maximal CPET based on RER; N/A = Not Applicable.

The results for the relative aerobic load of the five ADL are presented in **Table 4**. With the exception of P02 for descending stairs, all the ADL tasks in all participants were performed at relative aerobic load levels > 100% of ${\dot{V}O}_{2}$–VT_1_. The relative aerobic load (%${\dot{V}O}_{2}$–VT_1_) was highest in the Glittre ADL test and the TUGT. The Glittre ADL test evaluates functional capacity through a circuit of daily activities, including walking, going up and down a few steps, and moving objects. The modified version is identical but excludes the step component. In this study, there seems to be a difference in %${\dot{V}O}_{2}$–VT_1_ between participants performing the standard version and those completing the adjusted version without steps.

**Table 4:** % ${\dot{V}O}_{2}$–VT_1_ for the different ADL tasks.

Participant	01	02	03	04	05
**6MWT % ${\dot{V}O}_{2}$–VT_1_**	147	130	141	121	137
**TUGT % ${\dot{V}O}_{2}$–VT_1_**	237	150	202	278	168
**Glittre ADL test % ${\dot{V}O}_{2}$–VT_1_**	240	162^*^	166	225	157^*^
**Descending Stairs % ${\dot{V}O}_{2}$–VT_1_**	123	93	124	111	155
**Ascending Stairs % ${\dot{V}O}_{2}$–VT_1_**	170	105	137	174	118

* Adjusted Glittre ADL task without steps. Participant 06 is excluded.

**Figure 1*:**
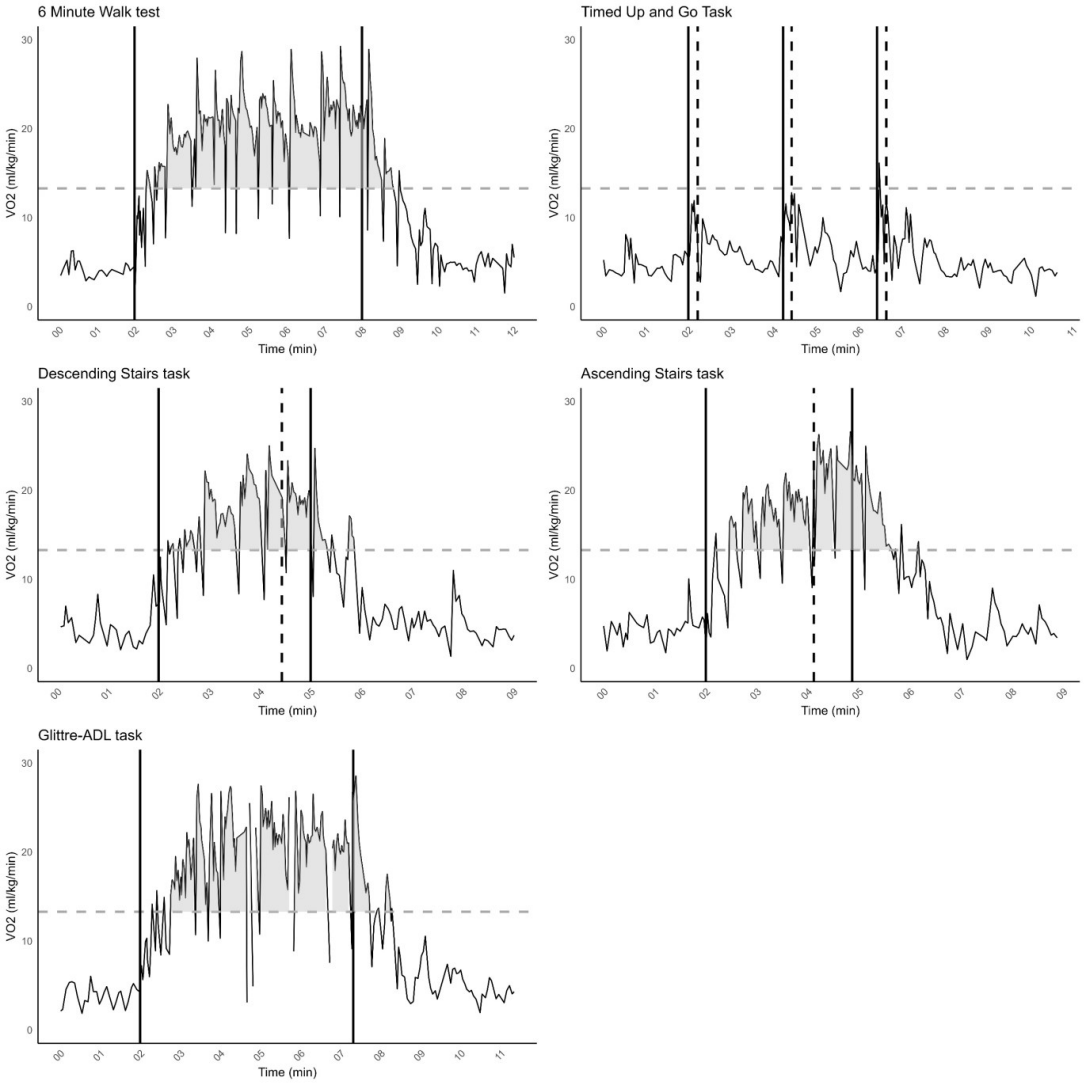
${\dot{V}O}_{2}$ curve for each ADL task for Participant 03. The dotted horizontal line is de ${\dot{V}O}_{2}$–VT_1_ of P03. The solid vertical lines represent the start and stop of the ADL task, respectively. In the TUGT the vertical solid line indicates moment of starting TUGT and the dashed vertical line is the end of the TUGT cycle. For both ascending and descending the stairs, the vertical dashed line indicates the moment of starting to climb the stairs.

To illustrate ${\dot{V}O}_{2}$ during each ADL task, **Figure 1** shows the measured ${\dot{V}O}_{2}$ curves for a randomly selected participant (P03). Data for the other participants are presented in **APPENDIX** 1–4.

* Figure 1 was corrected on March 26, 2026; see the published corrigendum for further details (https://doi.org/10.33137/cpoj.v9i1.47304).

**Figure 2:**
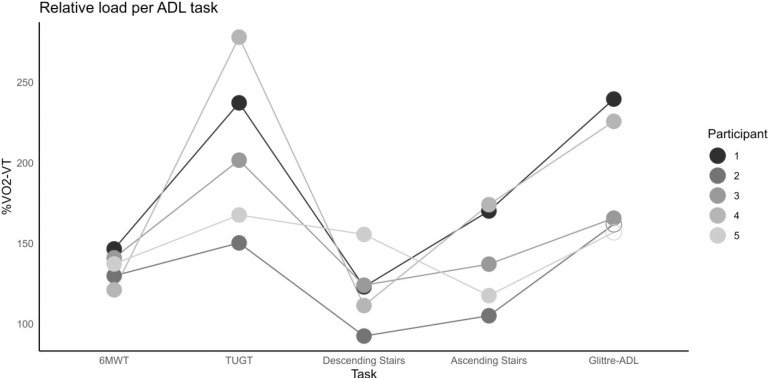
Relative aerobic load per ADL task. Scatterplot of the relative aerobic load for each participant on each test. Unfilled datapoints: Performed the adjusted version of the Glittre ADL test.

**Figure 2** shows the relative aerobic load (%${\dot{V}O}_{2}$–VT_1_) of each participant for each task, illustrating how participants rank across tasks. The ranking appears quite consistent over all tests. Variation between participants seems highest for the TUGT and lowest for the 6MWT. Using an ICC, the agreement in the ranking of the different tasks between participants was quantified (ICC = 0.73; 95% CI: 0.01–0.97).

## DISCUSSION

This study had two main objectives. First, we aimed to evaluate the developed protocol based on criteria related to operability, logistics, and suitability of intensity. Second, we examined whether the EPOC method, previously applied in individuals after stroke,^18^ can be used to determine the relative load of five ADL tasks of varying duration in individuals with LLA. The findings indicated that the protocol was feasible, demonstrated by successful implementation in five of six participants, with only minor adjustments required and one participant unable to complete the full protocol.

These results suggest that the protocol was operable and logistically manageable within the planned timeframe, and that the intensity of the tasks was appropriate for the target population. The need for small modifications, such as adapting Glittre ADL test for safety, highlights practical considerations for applying the protocol in clinical or research settings. Overall, the findings support the applicability of the protocol and confirm that the EPOC method can be used to quantify relative load during ADL tasks of varying duration in this population.

The Dutch population of individuals with LLA is highly heterogeneous, encompassing a wide range of comorbidities that may influence cardiorespiratory fitness. The participants in this study were a fair representation of this population,^21^ reflecting substantial variability in amputation level, etiology, and comorbid conditions. This diversity highlights the need for individualized assessment, as comorbidities such as cardiovascular disease, diabetes mellitus, and balance impairments can independently affect both cardiorespiratory fitness and functional capacity. For example, one participant was unable to perform the CPET due to difficulties in maintaining posture and movement on the one-legged cycling test, likely resulting from imbalance associated with LLA or comorbidities.

In terms of task intensity, the protocol was appropriate for most participants (5 out of 6). However, adaptations to the Glittre ADL test were necessary for three participants due to balance limitations and the use of walking aids to ensure safety. These adaptations involved replacing stair climbing with level walking over the same distance. This highlights the need for selecting functional ADL tests that can be uniformly administered across a diverse LLA population, particularly in research settings.

Regarding CPET outcomes, only one participant achieved a maximal effort as indicated by a respiratory exchange ratio (RER) > 1.1. The remaining participants did not achieve an RER > 1.1, suggesting that fatigue may have been driven by factors other than cardiopulmonary limitations. Five participants reached their first ventilatory threshold, supporting its use as a reliable reference point for determining relative aerobic load in this population. The mean ${\dot{V}O}_{2}$–VT_1_ (n = 5) was 10.0 ± 2.4 ml/kg/min. This is comparable to previously described ${\dot{V}O}_{2}$–VT_1_ in individuals with stroke^18^ with respective means of 9.7 (±2.4) ml/kg/min for functional ambulation category (FAC) 3, 9.9 (±2.7) ml/kg/min for FAC 4, and 10.9 (±2.8) ml/kg/min for FAC 5. The mean ${\dot{V}O}_{2}$peak achieved by the five participants was 13.1ml/kg/min (range 8.5–17.0). Note that this is the ${\dot{V}O}_{2}$peak obtained during the test, which was not considered a maximal test for 4 out of the 5 participants. Possibly these ${\dot{V}O}_{2}$peak values are therefore somewhat lower to those that were considered maximal in previous studies in dysvascular group of individuals with LLA, where mean ${\dot{V}O}_{2}$peak of 17.1 ± 4.1 ml/kg/min was reported. In individuals with traumatic LLA, the ${\dot{V}O}_{2}$peak values are reported to be higher (resp. 28.1 ± 6.7 ml/kg/min).^2^ In the study by Mellema et al.,^20^ the mean ${\dot{V}O}_{2}$peak was 21.8 ± 6.8 ml/kg/min, which falls between these previously reported ranges; however, this value was obtained using an arm-crank ergometer. Although differences in ${\dot{V}O}_{2}$peak likely reflect age, baseline fitness, and comorbidities, we did not analyze the association between aerobic capacity and functional performance (e.g., 6MWT distance). Future research should examine these relationships to clarify whether lower ${\dot{V}O}_{2}$peak corresponds to reduced mobility and endurance.

The ADL tasks in this study were selected to simulate ADL used in daily functioning and were aligned with previous findings on the ADL deemed most important by individuals with LLA.^19^ The Glittre ADL test which has recently been validated for individuals with LLA,^35^ served as a comprehensive measure of functional capacity. The relative aerobic load for the TUGT is challenging to interpret using the EPOC method as the time of action is quite short and error in the resting oxygen uptake level and the proportional impact of EPOC might have a large effect on the outcome (**Figure 2**). Consequently, it may require alternative methods or analyses for accurate assessment in these short-lasting ADL tasks.

In the remaining ADL tasks, the relative aerobic load determined with the EPOC method ranged between 100–200% of ${\dot{V}O}_{2}$–VT_1_, with the exception of P02 in the ADL descending stairs. These values are not unrealistic compared to data from Blokland et al.^18^ who applied the EPOC method in individuals post-stroke for tasks such as cycling, 5-minute walking, an obstacle course, sweeping leaves, and stair ambulation, reporting relative loads of 55–61% ${\dot{V}O}_{2}$peak for walking. This comparison suggests that the EPOC method provides realistic estimates of aerobic demand in individuals with LLA. Mellema et al.^20^ reported VO_2_ values of 11.4 ± 2.1 ml/kg/min for in-house walking and 22.5 ± 3.3 ml/kg/min for stair negotiation in 21 high-functioning individuals with LLA (14 transtibial and 7 transfemoral). Stair negotiation was performed at a self-selected speed of 0.55 ± 0.17 m/s compared to 0.69 ± 0.09 m/s in controls, despite similar VO_2_ values (22.5 ± 3.3 vs. 24.9 ± 3.4 ml/kg/min), illustrating that individuals with LLA expend nearly the same oxygen at a slower pace, indicating reduced gait economy. Importantly, the relative aerobic load was high: %${\dot{V}O}_{2}$peak averaged 55 ± 13% for in-house walking and exceeded 100% (102 ± 21%) for stair negotiation. With ${\dot{V}O}_{2}$-VT_1_ typically around 40–60% of ${\dot{V}O}_{2}$peak,^36^ these results align with our findings of 100–200% ${\dot{V}O}_{2}$-VT_1_ during the ADL tasks. Furthermore, the absolute VO_2_ values reported by Mellema et al. would translate into an even higher relative aerobic load in people with lower ${\dot{V}O}_{2}$peak, such as older adults or those with comorbidities, meaning that these activities could impose a substantially greater physiological burden. These findings highlight that the physiological burden during ADL is highly dependent on individual capacity. Since ${\dot{V}O}_{2}$peak and ${\dot{V}O}_{2}$-VT_1_ vary considerably between individuals with LLA, a generic level of physiological burden for each task cannot be given, instead it is essential to measure these parameters on a individual level to accurately determine relative load. Without such individualized assessment, there is a risk of underestimating or overestimating the actual exercise intensity, which has direct implications for rehabilitation planning and safety recommendations.

Interestingly, agreement in the ranking of relative aerobic load across ADL tasks among participants was observed (**Figure 2**). The point estimate of the ICC suggested moderate to good agreement (ICC = 0.73), but the very wide confidence interval (95% CI: 0.01–0.97) indicates substantial uncertainty around this estimate. Therefore, while the observed pattern may suggest that performance on one task could be indicative of aerobic demand in others, this finding should be interpreted cautiously and confirmed in a larger and more diverse sample.

### Strength and Limitations and Future Research

This study is one of the first to apply the EPOC method to assess the relative aerobic load of functional ADL tasks in individuals with LLA. The inclusion of both short and longer-duration tasks, such as stair climbing and the Glittre ADL test, demonstrates the method's applicability to non-steady-state activities that reflect daily functioning.

This study included individuals with K2–K3 ambulatory levels and reflected the clinical heterogeneity of the LLA population, including variation in amputation level, cause, and comorbidities. Although this variation underscores the importance of individualized assessment, since comorbidities and other factors may independently influence cardiorespiratory fitness and functional performance, the small sample size in this pilot study (six participants, five completed all tasks) limits generalizability. These findings should therefore be interpreted with caution, but they provide an initial indication of the importance of individualized testing.

Limitations include the small sample size, which restricts generalizability and subgroup analysis. Task feasibility was occasionally affected by environmental factors and individual safety concerns, requiring protocol adaptations. In addition, the wide confidence interval for the ICC (0.01–0.97) reflects the limited sample size and heterogeneity of participants, resulting in considerable uncertainty in this estimate. Therefore, these findings should be interpreted with caution, and future studies with larger cohorts are needed to provide more robust reliability estimates.

Future research should validate these findings in larger, more diverse samples and explore how rehabilitation interventions influence aerobic load and functional capacity. The use of wearable technology may further enable real-time, individualized monitoring of aerobic demand during ADL in daily life.

## CONCLUSION

The described protocol and selected functional ADL appear feasible for logistics and planning for K2–K3 individuals with LLA. This study demonstrates that shorter, non–steady-state ADL tasks can be measured in this population using the EPOC method. The protocol can be applied to estimate the relative aerobic load of functional ADL, offering preliminary insights into the capabilities and challenges faced by individuals with LLA. However, these findings should be interpreted with caution due to the small sample size and the need for task adaptations in some participants. While the EPOC method shows promise, its suitability for very short-burst activities remains uncertain, as these may not allow sufficient time for accurate measurement. Further research is needed to confirm its applicability in such contexts and to validate the protocol in larger and more diverse cohorts before broad generalization. Importantly, the observed variability in amputation level, physical capacity, and comorbidities underscores the importance of individualized assessment. Identifying the specific aerobic demands of ADL at the individual level may support the development of individualized rehabilitation programs tailored to the capabilities and limitations of each individual. Such an approach could facilitate more realistic goal-setting, improve fatigue management, and ultimately enhance participation and quality of life for individuals with LLA.

## Figures and Tables

**Figure:**
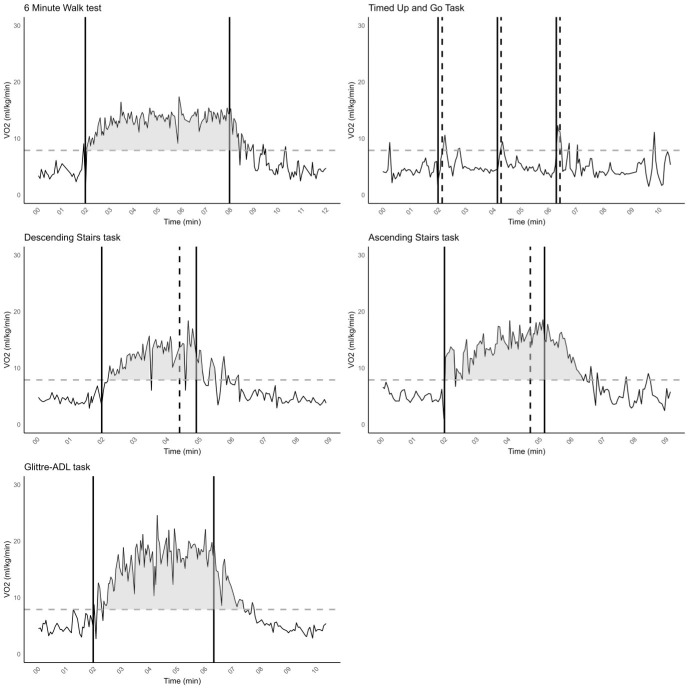


**Figure:**
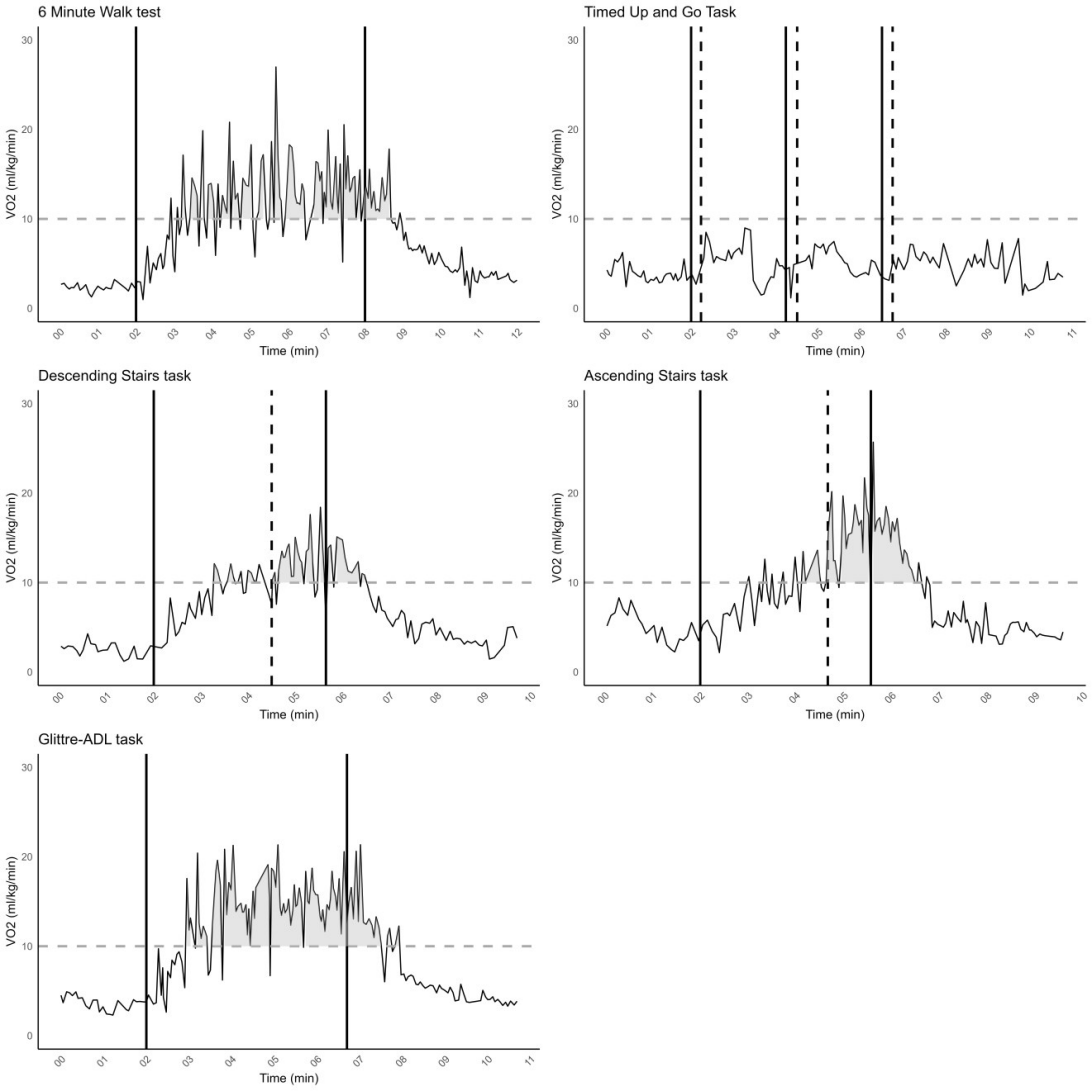


**Figure:**
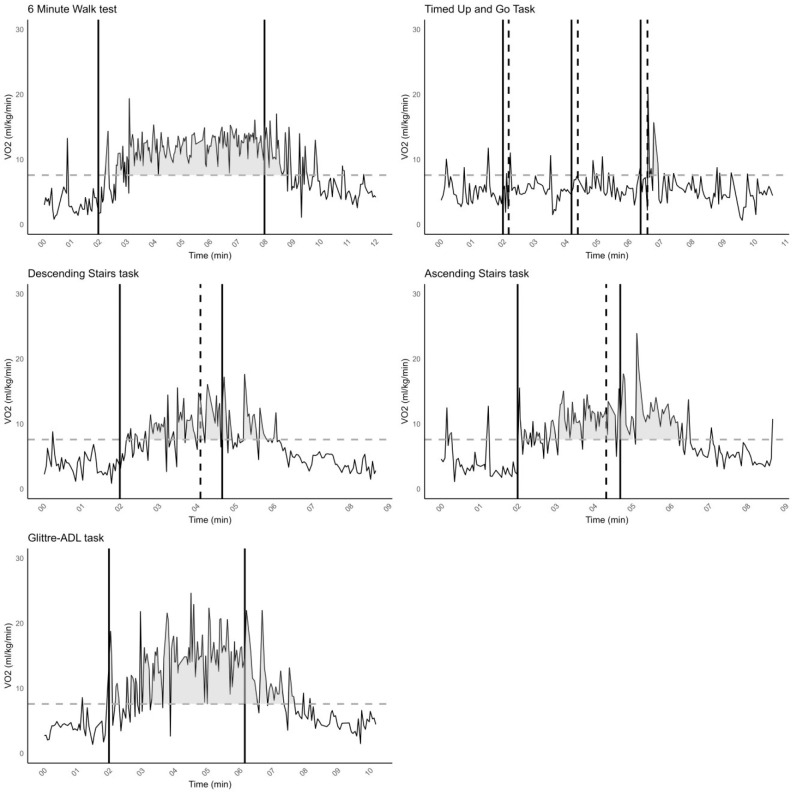


**Figure:**
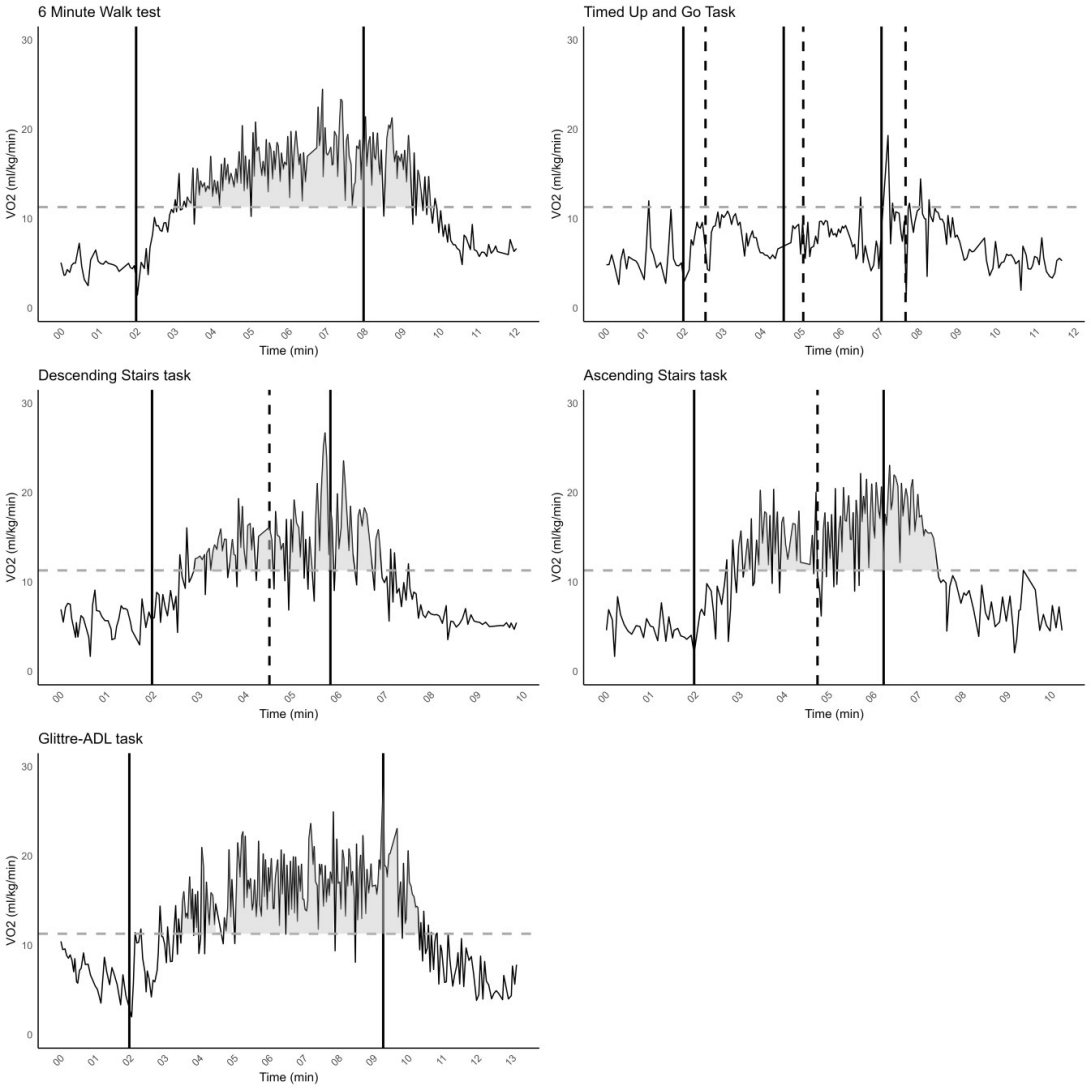


## Footnotes

# Ergometer: ergoselect 200 (Ergoline); CPX: Vyntus CPX Professional (Vyaire); ECG: Cardiosoft 6; Blood pressure monitor: Tango M2 (Suntech).

## APPENDIX

${\dot{V}O}_{2}$ curve for each ADL task for Participant 1, 2, 4 and 5. The dotted horizontal line is the ${\dot{V}O}_{2}$-VT_1_ of P01. The solid vertical lines represent the start and stop of the ADL task, respectively. In the TUGT the vertical solid line indicates moment of starting TUGT and the dashed vertical line is the end of the TUGT cycle. For both ascending and descending the stairs, the vertical dashed line indicates the moment of starting to climb the stairs

### Appendix 1

Participant 01

### Appendix 2

Participant 02

### Appendix 3

Participant 04

### Appendix 4

Participant 05
