# Corrigendum: Relative aerobic load of daily activities in individuals with lower limb amputation: A pilot study

**DOI:** 10.33137/cpoj.v9i1.47304

**Published:** 2026-03-26

**Authors:** L van Schaik, K van Kammen, S.W.M Huiberts, J.H.B Geertzen, H Houdijk, R Dekker

**Affiliations:** 1 University of Groningen, University Medical Center Groningen, Department of Rehabilitation Medicine, Groningen, The Netherlands.; 2 University of Groningen, University Medical Center Groningen, Department of Human Movement Sciences, Groningen, The Netherlands.

In the article “Relative aerobic load of daily activities in individuals with lower limb amputation: A pilot study,” published in Canadian Prosthetics & Orthotics Journal, Volume 8, Issue 2, 2025 (https://doi.org/10.33137/cpoj.v8i2.46249),^1^ an error was identified in Figure 1. The graphical representation of the progression of VO_2_ relative to VT1 was not displayed correctly. This issue concerns the visualization only; all underlying data, analyses, and remaining figures are accurate, and the study's results and conclusions remain unchanged. The corrected version of Figure 1 has now been provided. The authors apologize for this oversight.

**Figure 1 (corrected):**
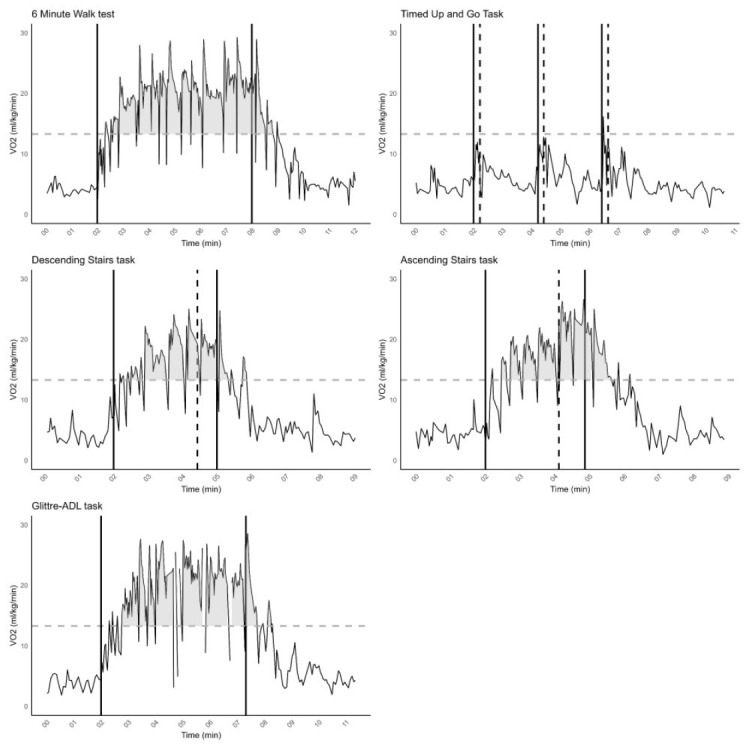
${\dot{V}O}_{2}$ curve for each ADL task for Participant 03. The dotted horizontal line is de ${\dot{V}O}_{2}$–VT_1_ of P03. The solid vertical lines represent the start and stop of the ADL task, respectively. In the TUGT the vertical solid line indicates moment of starting TUGT and the dashed vertical line is the end of the TUGT cycle. For both ascending and descending the stairs, the vertical dashed line indicates the moment of starting to climb the stairs.
